# Hydrothermal Synthesis and Optical Properties of Magneto-Optical Na_3_FeF_6_:Tb^3+^ Octahedral Particles

**DOI:** 10.3390/ma13020320

**Published:** 2020-01-10

**Authors:** Zhiguo Zhao, Xue Li

**Affiliations:** 1 Key Laboratory of Electromagnetic Transformation and Detection of Henan province, Luoyang Normal University, Luoyang 471934, China; 2 School of Materials Science and Engineering, Zhejiang Sci-Tech University, Xiasha University Town, Hangzhou 310018, China

**Keywords:** magnetic-optical bi-functional materials, hydrothermal process, down-conversion luminescence, Na_3_FeF_6_:Tb^3+^

## Abstract

Sodium iron hexafluoride (Na_3_FeF_6_), as a colorless iron fluoride, is expected to be an ideal host for rare earth ions to realize magneto-optical bi-functionality. Herein, monodispersed terbium ions (Tb^3+^) doped Na_3_FeF_6_ particles are successfully synthesized by a facile one-pot hydrothermal process. X-ray diffraction (XRD) and Field emission scanning electron microscopy (FESEM) reveal that the Tb^3+^ doped Na_3_FeF_6_ micro-particles with regular octahedral shape can be assigned to a monoclinic crystal structure (space group P21/c). Under ultraviolet light excitation, the Na_3_FeF_6_:Tb^3+^ octahedral particles given orange-red light emission originated from the ^5^D_4_→^7^F_J_ transitions of the Tb^3+^ ions. In addition, the magnetism measurement indicates that Na_3_FeF_6_:Tb^3+^ octahedral particles are paramagnetic with high magnetization at room temperature. Therefore, the Na_3_FeF_6_:Tb^3+^ powders may find potential applications in the biomedical field as magnetic-optical bi-functional materials.

## 1. Introduction

Bi-functional materials with distinct magnetic and fluorescent (luminescent) properties have received considerable attention [1,2] due to their potential applications in magnetic resonance imaging [3], targeted drug delivery [4], sensors [5,6], optical isolators [7,8,9], high accuracy communication [10], and aircraft guidance [11]. To date, there have been a few reports about the synthesis of magnetically-functionalized luminescent materials based on quantum dots (QDs) and organic dyes [12]. However, QDs features notorious disadvantages including chemical instability, potential toxicity, luminescent intermittence and weakly magnetic, while organic dyes typically exhibit rapid photobleaching and a low fluorescence quantum yield [13]. As a result, biological applications of these materials have been seriously restricted.

Rare-earth (RE) ions doped inorganic materials can be considered as alternative luminescent materials in which the above limitations are partly circumvented [14,15]. Nowadays, great efforts have been devoted to the design and fabrication of magneto-optical bi-functional systems based on RE-doped up-conversion or down-conversion materials, such as Gd_2_O_3_: Er^3+^/Yb^3+^ [16], GdPO_4_: Eu^3+^ [17], YVO_4_: Er^3+^ [18], Tb_0.94_Pr_0.06_VO_4_ [19] and NaYF_4_: Yb, Ho [20]. However, studies of magneto-optical effects usually have to rely on materials with a high magnetic movement, which are usually non-transparent. On the other hand, the introduction of strong magnetic (ferromagnetic) materials can be achieved by fabricating magnetic-core/luminescent-shell structures, such as the Fe_3_O_4_@LaF_3_:Yb^3+^, Er^3+^ [21], Fe_3_O_4_@α-NaYF_4_/Yb [13] and Fe_3_O_4_@ZnO:Er^3+^,Yb^3+^ [22]. However, the preparation processes for core-shell structures is complicated, and more importantly, magnetic oxide, Fe_3_O_4_, strongly absorbs visible light and quenches fluorescence of the RE ions [23]. Therefore, use of a colorless, strongly magnetic host is of great importance for the development of magneto-optical bifunctional materials.

In this work, colorless Tb^3+^ ions doped sodium iron hexafluoride (Na_3_FeF_6_:Tb^3+^) containing a high centration of paramagnetic ion (Fe^3+^) is synthesized through a simple hydrothermal process. The Na_3_FeF_6_:Tb^3+^ particles give distinct visible emission under excitation by UV light and its luminescence intensity is optimized by adjusting Tb^3+^ doping concentration. The investigation of the magnetic property reveals that the Na_3_FeF_6_:Tb^3+^ particles are paramagnetic at room temperature. These results indicate that Na_3_FeF_6_:Tb^3+^ particles might be promising as a new platform for exploiting magnetic-optical functionalities.

## 2. Materials and Methods 

### 2.1. Synthesis of Na_3_FeF_6_:Tb^3+^ Particles

The reagents used in this work were analytical-grade Fe(NO_3_)_3_·9H_2_O (99.99%), NH_4_HF_2_ (99%), NaF (99%), HF (40%), and Tb(NO_3_)_3_·6H_2_O (99.99%) (Xiya Reagent, Shandong, China). Samples with a different molar ratio of Tb^3+^ to Fe^3+^ (5%, 10%, 15%, 18%, and 20%) were synthesized by a hydrothermal method under the same conditions. Here, we take Na_3_FeF_6_:18%Tb^3+^ as an example to present the detailed preparation procedure. The process mainly involves four steps: (1) 14 mL of Fe(NO_3_)_3_·9H_2_O solution (0.1 M), 14 mL of NaF solution (0.5 M), 42 mL of NH_4_HF_2_ solution (0.5 M), and 3 mL of HF were mixed under vigorous magnetic stirring for 30 min; (2) 2.7 mL Tb(NO_3_)_3_·6H_2_O was added to the above solution under vigorous magnetic stirring for 3 h; (3) after stirring for 3 h, the above solution was transferred into a Teflon-lined stainless steel autoclave (capacity 100 mL), which was heated at 190 °C for 12 h and cooled naturally to room temperature; (4) the obtained sample was washed by deionized water for several times and dried at 60 °C overnight.

### 2.2. Characterization

Phase identification of the as-prepared samples were carried out by X-ray diffraction (XRD) (X’Pert Pro, PANalytical BV, Netherland) with Cu Kα radiation (λ = 1.5418 Å). The microstructure and element mapping of particles were observed with a Field emission scanning electron microscopy (FESEM) (Hitachi Ltd., Tokyo, Japan) equipped with an energy dispersive spectroscopy (EDS). UV-Vis (ultraviolet-visible) absorption, transmission, and reflectance spectra of particles were acquired in an UV-Vis spectrophotometer (Model: U3600P) with an integrating sphere using BaSO_4_ as a standard reference. Photoluminescence excitation and emission spectra were obtained using two spectrometers (Omni-λ3007 and Omni-λ180D; Zolix, Beijing, China) and a 150 W Xenon lamp as the excitation source. The Commission International de I’Eclairage (CIE) chromaticity coordinates of sample were calculated by CIE 1931 software (V.1.6.0.2). Magnetic properties were collected on a Quantum Design superconducting quantum interference device (SQUID) magnetometer (MPMS XL-7).

## 3. Results and Discussion

**Figure 1:**
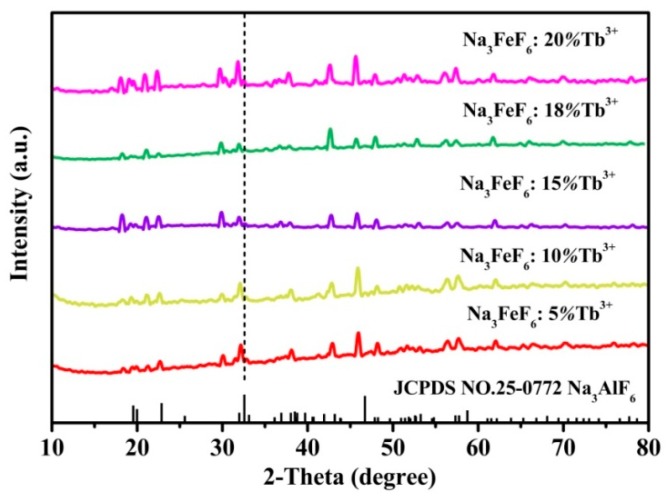
X-ray diffraction (XRD) patterns for samples of the Na_3_FeF_6_:Tb^3+^ with different Tb^3+^-doping concentrations.

**Figure 2:**
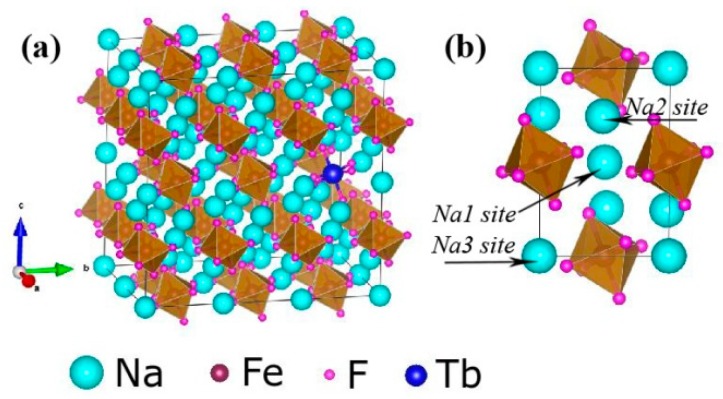
(**a**) Three-dimensional crystal structure of Na_3_FeF_6_:Tb^3+^. (**b**) Three different sodium sites in the Na_3_FeF_6_ crystal structure.

Figure 1 shows the typical XRD patterns of the Tb^3+^ doped Na_3_FeF_6_ samples synthesized with different doping concentrations of Tb^3+^ (5%, 10%, 15%, 18%, and 20%). The diffraction peaks of all samples clearly match that of the standard pattern of Na_3_AlF_6_ (JCPDS no. 12-0907), indicating the structure of obtained samples is isomorphic with cryolite-like structures (Na_3_AlF_6_ and Na_3_CrF_6_) that belongs to the space group P21/c [24,25,26]. This result agrees with previous report about the structure of Na_3_FeF_6_ [27]. The three-dimensional crystal structure of Na_3_FeF_6_:Tb^3+^ is shown in Figure 2. There are three different sodium sites, namely Na1, Na2, and Na3, as highlighted in Figure 2b. Na1 site is located at the distorted octahedral site of (NaF_6_), Na2 site is located at the bi-pyramid site of (NaF_5_), and the Na3 site is located at the distorted tetrahedral site of (NaF_4_). As can be observed from the crystal structure (Figure 2a), Na1 octahedral and Na3 tetrahedral share corners. Na1 octahedral share edges with Na2 bipyramid. Furthermore, all Fe atoms are located at the distorted FeF_6_ octahedral sites. FeF_6_ octahedra share corners with Na1 octahedral and Na3 tetrahedral share edges with Na2 bi-pyramid. In this structure, Fe^3+^ sites can be taken by Tb^3+^ ions in Tb doped Na_3_FeF_6_. According to the Bragg equation (2dSinθ = nλ), d increases with the decreasing of θ. Figure 1 shows that diffraction peak of Na_3_FeF_6_:Tb^3+^ is all shifted to the left compared with that of Na_3_AlF_6_ due to the larger ionic size of Fe^3+^ as compared with that of Al^3+^. As concentration of the Tb ions increases from 5% to 18%, the diffraction peak gradually shifts to the left, diffraction angle θ decreases. This result can be explained by the substitution of Fe^3+^ (ionic radius = 0.65 Å) [28] by Tb^3+^ with a larger ionic radius (0.92 Å) [29]. Therefore, the lattice constant would increase with the increase in the concentration of Tb ions in the lattice. The diffraction peaks of the Na_3_FeF_6_ with 18% Tb^3+^ doping are the highest, indicating the best crystallinity. The increase of Tb^3+^ concentration above 18% leads to growth of lattice strain that prevents the further enhancement of crystallization. To further confirm the ions of Tb^3+^ is present in the form of Tb-F and Na_3_FeF_6_:18%Tb^3+^ powder was analyzed by XPS (Figure S1). The XPS spectrum shows the presence of Na, F, Fe and Tb elements. Figure S1b shown the XPS spectra of Tb(Ds-4s), Na(2p), F(2s), Fe(3p), Tb(4d) from Na_3_FeF_6_:18%Tb^3+^ and the relatively strong peaks at around 7.5, 152 eV can be assigned to the binding energy of Tb (Ds-4s) and Tb (4d), respectively. The peak around 24.7 eV is attributed to the binding energy of Na(2p). The binding energy of F(2s) around 30.3 eV and F(1s) around 684.9 eV are found in spectra of XPS (Figure 1b,d). The Fe(3p) peaks show a doublet around 56.3 and 59.1 eV, corresponding to structure of FeF_3_ and FeF_2_, respectively. The result in accordance with the discussion of the XRD patterns of the Tb^3+^ doped Na_3_FeF_6_.

**Figure 3:**
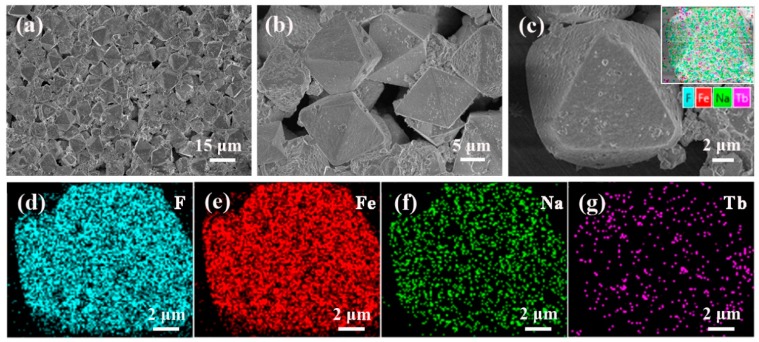
(**a**–**c**) Field emission scanning electron microscopy (FESEM) images of the Na_3_FeF_6_:18%Tb^3+^ powders and (**d**–**g**) the corresponding energy dispersive spectroscopy (EDS) mapping for elements image F, Fe, Na, and Tb.

The Na_3_FeF_6_:18%Tb^3+^ particles are then observed by FESEM equipped with an energy dispersive spectroscopy (EDS) device. Figure 3a–c show the FESEM images with low magnification (a) and high magnification (b,c). It can be observed from Figure 3a that the as-prepared samples consist of randomly distributed octahedral particles with a relatively uniform size and shape (edge lengths are approximately 10 µm). As the magnification increases (Figure 3c), it can be seen clearly that the surfaces of the octahedron are almost smooth, but covered by a few small sized particles. EDS analysis was then used to determine the distribution of elements, as illustrated in Figure 3c. The results confirm the dominance of four elements:F, Fe, Na and Tb. In addition, the corresponding EDS mapping images given in Figure 3d–g reveal that all the elements are distribute homogeneously in the particles and Tb ions are successfully doped into the lattice of Na_3_FeF_6_.

**Figure 4:**
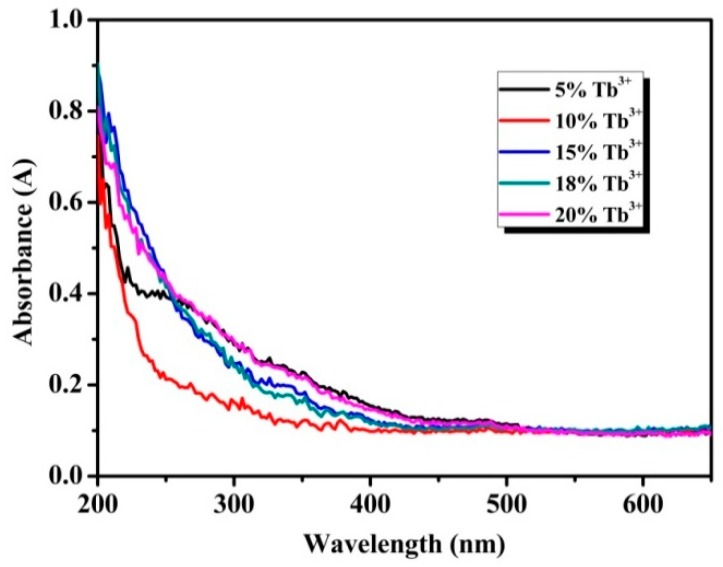
Ultraviolet-visible (UV-Vis) absorption spectra of Na_3_FeF_6_:Tb^3+^ particles with different Tb^3+^-doping concentrations.

To confirm the optical response of the particles in the UV-Vis range, absorption spectra was detected by an UV-Vis spectrophotometer. As shown in Figure 4, all samples exhibit obvious ultraviolet absorption at wavelength short than 300 nm, which can be attributed to transition of the 4f electronic ground state to the 5d energy levels, namely ^4^f_8_→^4^f_7_^5^d_1_ energy levels transitions of Tb^3+^ [30]. The f-f transitions of the Tb^3+^ in the wavelength region of 300-400 nm are relatively weak and these peaks at 355 and 380 nm by f-f transitions of Tb^3+^ are almost invisible in the absorption spectra [31]. The transmission spectra and the reflectance spectra of the Na_3_FeF_6_:18%Tb^3+^ particles correspond to the absorption spectra (as shown in Figure S2).

**Figure 5:**
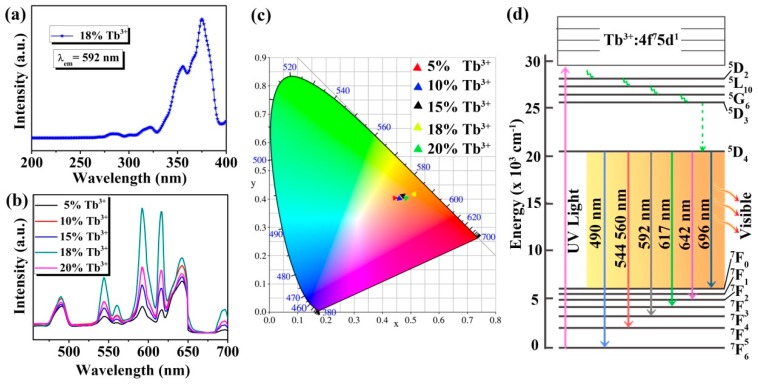
(**a**) Excitation spectrum of Na_3_FeF_6_:18%Tb^3+^; (**b**) emission spectra of Na_3_FeF_6_:Tb^3+^ particles with different Tb^3+^-doping concentrations; (**c**) CIE 1931 chromaticity diagram of Na_3_FeF_6_ doped with different concentration of Tb^3+^; (**d**) simplified energy levels diagram of the Tb ions.

In order to further study the optical properties of the Na_3_FeF_6_:Tb^3+^ particles, excitation and emission spectra are measured by fluorescence spectrometers. Figure 5 presents the excitation and emission spectra, and CIE 1931 chromaticity coordinates of the samples together with the energy level diagram of Tb ions. As shown in Figure 5a, the excitation spectra of Na_3_FeF_6_:18%Tb^3+^ are measured for the emission wavelength of 592 nm. It can be observed that the excitation spectra consist of sharp and intense bands with peak positions at 355 and 375 nm along with weak bands at 280 and 320 nm, which can be assigned to the ^7^F_6_→^5^L_10_, ^7^F_6_→^5^G_6_, ^7^F_6_→^3^H_6_ and ^7^F_6_→^5^D_1_ transitions of the Tb^3+^, respectively [32,33,34].

Since the peak of 375 nm is the strongest in the excitation spectrum, the emission spectra are recorded at this excitation wavelength for Na_3_FeF_6_:Tb^3+^ particles with different Tb^3+^-doping concentrations. As can be seen from Figure 5b, the emission spectra in the region of 455–700 nm exhibit seven peaks at 490, 544, 560, 592, 617, 642, and 696 nm due to ^5^D_4_→^7^F_6_, ^7^F_5_, ^7^F_5_, ^7^F_4_, ^7^F_3_, ^7^F_2_ and ^7^F_0_ transitions, respectively [35,36,37,38]. Among the seven peaks, five peaks at 490, 544, 592, 617, and 642 nm are much stronger, while the other two peaks (560 and 696 nm) are relatively weak. In addition, among the five samples, the luminescence intensity is strongest when the doping concentration of Tb^3+^ is 18%, and the highest luminescence peak is at 592 nm. The emission spectrum is converted to the CIE 1931 chromaticity coordinates using the photoluminescence data to better characterize the emission color of the samples. From the CIE 1931 chromaticity diagram (Figure 5c), it is found that all samples emit orange-red light, which is different from the traditional green light emission of Tb^3+^ ions. This may be due to the use of a new host (Na_3_FeF_6_) which favors the emission in the longer wavelengths. Furthermore, Figure 5c shows that as the doping concentration of Tb ions increases, the luminescence intensity first increases and then decreases, and the luminescence is strongest at the doping concentration of 18%, which is consistent with the emission spectrum (Figure 5b). The CIE coordinates of Na_3_FeF_6_:18%Tb^3+^ are X = 0.5103 and Y = 0.4155, which show a typical orange-red color.

In order to better understand the luminescence mechanism of the samples, we combined the energy level diagram of Tb ions (Figure 5d) and take the luminescence at 592 nm as an example to explain the involved electronic transitions. Upon excitation by ultraviolet light (UV-light), Tb^3+^ ions are promoted from the ground state (^7^F_6_) to the excited state (for example ^5^L_10_, ^5^G_6_). Subsequently, the level ^5^D_4_ of Tb^3+^ ions is populated by radiation-free transition. Finally, the Tb^3+^ ions relax to the ground state (^7^F_4_) by giving visible emission at around 592 nm. The visible luminescence at other wavelengths is similar to the emission at 592 nm.

**Figure 6:**
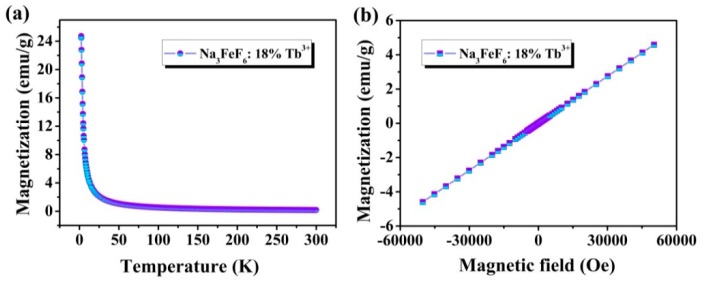
(**a**) Temperature-dependent magnetization (M-T) curves measured at 2000 Oe for Na_3_FeF_6_:18%Tb^3+^ particles; (**b**) magnetization versus magnetic field (M-H) curve at 300 K of Na_3_FeF_6_:18%Tb^3+^ particles.

Figure 6a shows the temperature-dependence magnetization plots (M-T) in a temperature range between 5 K and 300 K in a 2000 Oe field of Na_3_FeF_6_:Tb^3+^ particles. It is found that the magnetization decreases rapidly from about 24.74 emu/g at 5 K to 1.06 emu/g at 50 K, and then slowly decreases with a temperature increase from 50 K to 300 K, typical for paramagnetic materials. The magnetization versus magnetic field (M-H) curves at 300 K of Na_3_FeF_6_:18%Tb^3+^ particles obtained by SQUID magnetometry are presented in Figure 6b. As the strength of the applied magnetic field increasing, the ideal linear correlation between the magnetization and the applied magnetic field was obtained, indicating that Na_3_FeF_6_:Tb^3+^ possesses paramagnetism. The results show that the synthesized samples might be used as magneto-optical bifunctional materials.

## 4. Conclusions

In summary, monodispersed Na_3_FeF_6_:Tb^3+^ octahedral particles have been successfully synthesized by a facile one-pot hydrothermal process and the results of XRD and SEM indicated that Na_3_FeF_6_:Tb^3+^ octahedral belong to a monoclinic crystal structure (space group P21/c). The Na_3_FeF_6_:Tb^3+^ octahedral particles emit orange-red colored light attributed to the ^5^D_4_ → ^7^F_J_ transitions of the Tb^3+^ ions. The luminescence intensity of the Na_3_FeF_6_:Tb^3+^ reaches maximum at Tb^3+^ doping concentration of 18%. The M-T and M-H curves confirm that Na_3_FeF_6_:Tb^3+^ particles are paramagnetic with a high magnetic moment. These results indicate that the obtained Na_3_FeF_6_:Tb^3+^ octahedral particles might be used as a magnetic-optical bi-functional material for various potential applications in biomedical fields and magneto-optical modulation.

## Supplementary Materials

The following are available online at https://www.mdpi.com/1996-1944/13/2/320/s1.
