# Supplementary material for: Hydrothermal Synthesis and Optical Properties of Magneto-Optical Na3FeF6:Tb3+ Octahedral Particles
Source: Materials (Basel). 2020 Jan 10;13(2):320. doi: 10.3390/ma13020320 (PMC7013557; doi:10.3390/ma13020320)
Supplement: Supplementary file 1 [file materials-13-00320-s001.pdf]

materials

MDPI

Hydrothermal Synthesis and Optical Properties of Magneto-Optical $Na_3FeF_6:Tb^{3+}$ Octahedral Particles

Zhiguo Zhao [1,*] and Xue Li [2,*]

[1] Key Laboratory of Electromagnetic Transformation and Detection of Henan province, Luoyang Normal University, Luoyang 471934, China

[2] School of Materials Science and Engineering, Zhejiang Sci-Tech University, Xiasha University Town, Hangzhou 310018, China

* Correspondence: zhiguo.zhao@tom.com (Z.Z.); lixue5306@163.com (X.L.)

Z.Z. and X.L. contributed equally.

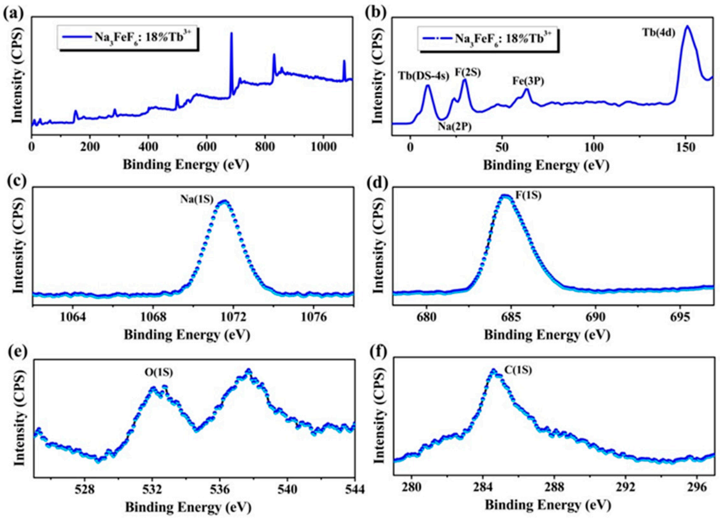

Figure S1. XPS spectra of the Na_3FeF_6:18%Tb^{3+} powders

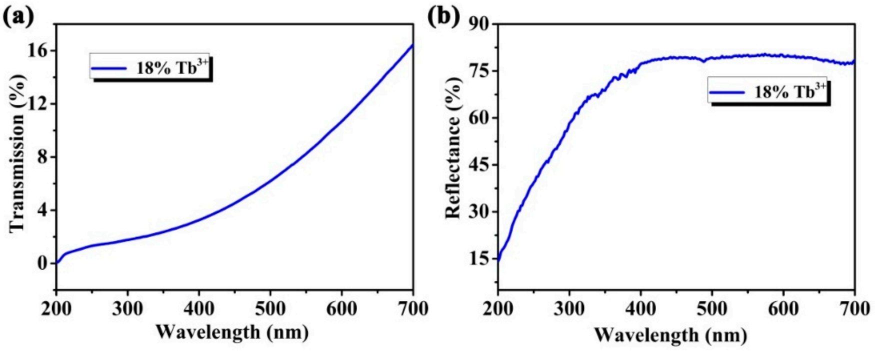

Figure S2. (a) Transmission spectra and (b) reflectance spectra of Na_3FeF_6: 18%Tb^{3+} powders

© 2020 by the authors. Submitted for possible open access publication under the terms and conditions of the Creative Commons Attribution (CC BY) license (http://creativecommons.org/licenses/by/4.0/).
